# Coronavirus Disease 2019 (COVID-19) Infection Associated With Antiphospholipid Antibodies and Four-Extremity Deep Vein thrombosis in a Previously Healthy Female

**DOI:** 10.7759/cureus.8408

**Published:** 2020-06-02

**Authors:** Joowhan Sung, Seher Anjum

**Affiliations:** 1 Internal Medicine, MedStar Southern Maryland Hospital, Clinton, USA; 2 Translational Mycology, National Institute of Allergy and Infectious Diseases, Bethesda, USA

**Keywords:** covid-19, sars-cov-2, deep vein thrombosis, thromboembolism, antiphospholipid antibody

## Abstract

Infection caused by novel coronavirus (severe acute respiratory syndrome coronavirus 2, SARS-CoV-2) has been associated with coagulopathy. We present a case of a previously healthy 49-year-old female who was admitted to the hospital for coronavirus disease 2019 (COVID-19) pneumonia and later found to have extensive deep vein thrombosis (DVT) in all four extremities. This was accompanied by a steep rise in D-dimer levels and positive antiphospholipid antibodies (APLA) on further testing. She clinically improved on hydroxychloroquine and therapeutic anticoagulation. This is one of the first case reports describing APLA-associated DVT in a patient with COVID-19 pneumonia. Transient elevation of APLA from the viral illness may play a role in thrombosis associated with COVID-19.

## Introduction

Severe acute respiratory syndrome coronavirus 2 (SARS-Cov-2), a novel respiratory coronavirus, was first reported in December 2019 in Wuhan, China with subsequent global spread. As of May 24, 2020, a total of 5,335,868 confirmed cases and 341,549 deaths from coronavirus disease 2019 (COVID-19) were reported worldwide [1]. While the pathophysiology of COVID-19 infection remains poorly understood, coagulopathy is commonly observed and higher mortality has been reported in patients with elevated D-dimer levels [2-4]. Here we report a novel case of COVID-19 in a previously healthy patient who was complicated by extensive deep vein thrombosis (DVT) in all four extremities.

## Case presentation

**Figure 1:**
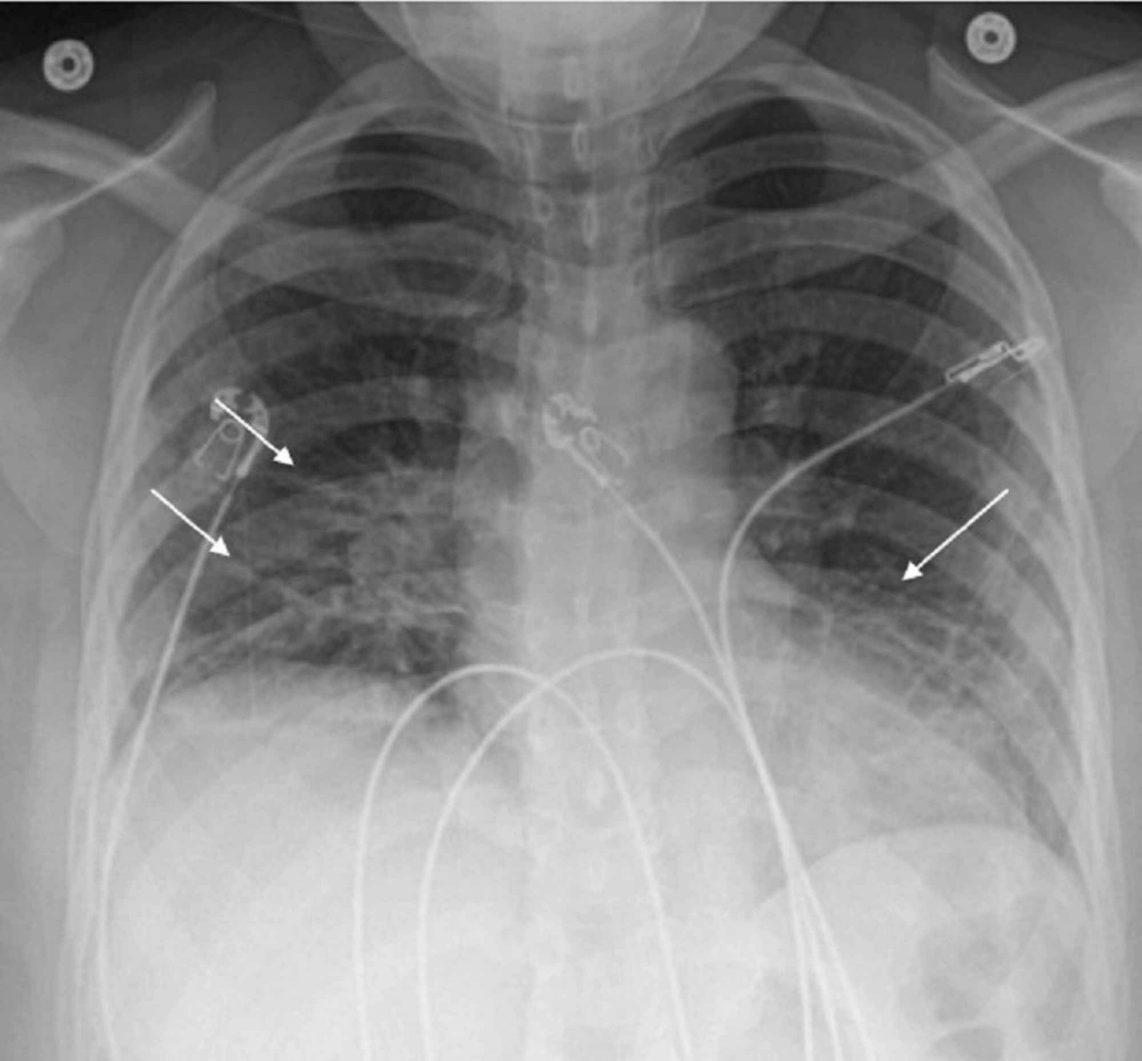
Chest X-ray on admission showing bilateral interstitial infiltrates predominantly in the lower lung fields.

A 49-year-old African American female presented to the emergency room with fever, cough, and myalgia in March 2020. She was obese (BMI of 36), but otherwise a previously healthy non-smoker who worked at a local grocery store in the suburbs of Washington, DC. Five days prior to presentation, she developed a cough, runny nose, and loss of appetite. This was followed by subjective fevers and progressive shortness of breath. On arrival to the emergency room, her temperature was 37.9°C, she was tachypneic with a respiratory rate of 31 breaths/min, tachycardic with a heart rate of 115 beats/min, and blood pressure was 111/81 mmHg. Her oxygen saturation on room air was 87%. Laboratory workup showed while blood cell count (WBC) of 8,400/μL, hemoglobin of 13.8 gm/dL, platelet of 257,000/μL. C-reactive protein (CRP) was 153 mg/L, ferritin was 148 ng/mL, international normalized ratio (INR) was 1.1, activated partial thromboplastin time (aPTT) was 31.0 seconds, fibrinogen was 542 mg/dL and D-dimer was 0.80 mcg/mL. Chest X-ray revealed bilateral interstitial infiltrates predominantly in the lower lung fields (Figure 1). She was started on intravenous (IV) ceftriaxone and azithromycin for concerns of community-acquired pneumonia. COVID-19 real-time reverse-transcription polymerase chain reaction (RT-PCR) from nasopharyngeal swab was positive and influenza rapid test was negative.

**Figure 2:**
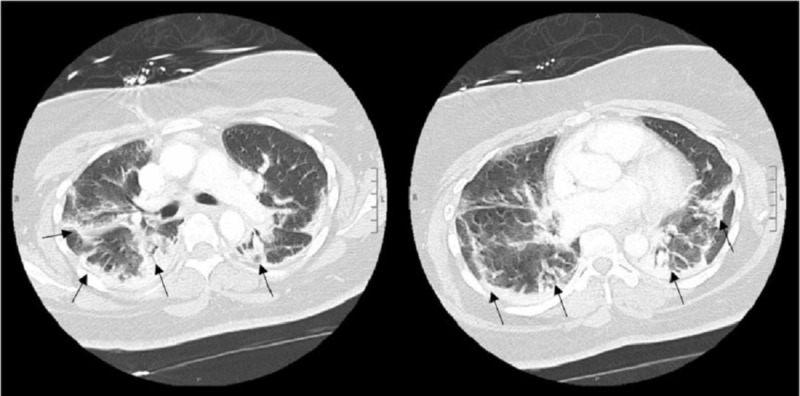
Chest CT on hospital day 2 showing peripheral patchy airspace opacities.

A chest computed tomography (CT) performed with contrast showed peripheral patchy opacities predominantly in the right upper lobe and the superior segments of the lower lobes but without evidence of pulmonary embolism (Figure 2). She required five to six liters of supplemental oxygen for persistent hypoxia. 

**Figure 3:**
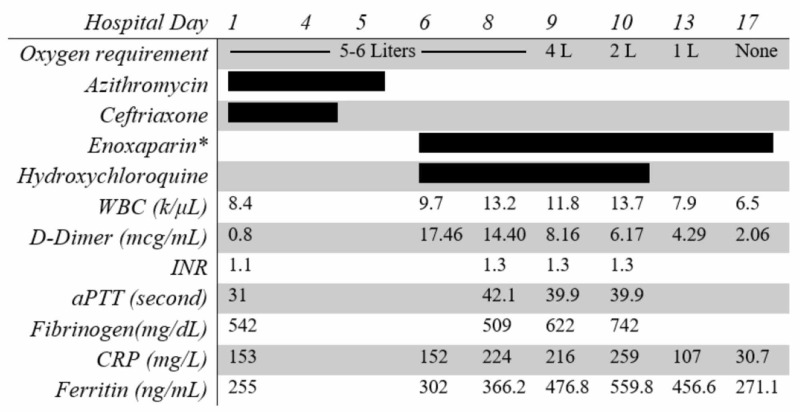
Timeline for oxygen requirements, treatments received, and laboratory parameters. *Systemic anticoagulation with 1 mg/kg every 12 hours. WBC, while blood cell count; INR, international normalized ratio; aPTT, activated partial thromboplastin time; CRP, C-reactive protein.

On hospitalization day 6, she was noted to have bilateral upper arm swelling at prior peripheral IV catheter insertion sites. Doppler showed occlusive thrombi in the brachial and cephalic veins bilaterally. D-dimer at the time was 17.46 mcg/mL, significantly increased from 0.80 mcg/mL on admission. The platelet count was 250,000/μL, INR was 1.3, and fibrinogen was 509 mg/dL. Other inflammatory markers including CRP and ferritin remained similar to prior (Figure 3). She was started on therapeutic anticoagulation with low molecular weight heparin (enoxaparin) 1 mg/kg every 12 hours. She was simultaneously started on hydroxychloroquine (400 mg PO twice a day for one day, followed by 200 mg twice a day for four days). On the following day, she complained of left calf pain and was noted to have tenderness on palpation of the area. Doppler showed bilateral occlusive thrombi in the popliteal veins and nonocclusive thrombi in bilateral lower femoral and right peroneal veins. Apart from obesity, she did not have any other personal risk factors or familial history of thromboembolism. Hypercoagulability workup was pursued; lupus anticoagulant was positive by dilute Russell viper venom time (dRVVT) but negative by platelet neutralization procedure (PNP). Both IgG (71 IgG phospholipid units, normal range ≤ 14) and IgM (39 IgM phospholipid units, normal range ≤ 15) anticardiolipin antibodies were elevated. IgA anticardiolipin antibody was within normal limits (6 IgA phospholipid units, normal range ≤ 11). Beta-2 glycoprotein IgM, IgG, and IgA were within the normal range. On hospital day 8, she was noted to have chest pain with tachycardia, which later resolved spontaneously. On hospital day 9, after three days of anticoagulation and hydroxychloroquine therapy, she was noted to have significant improvement in respiratory status and swelling of extremities. Her oxygen requirements decreased gradually and on hospital day 17 she was weaned off oxygen. She was subsequently discharged home.

## Discussion

Coagulopathy is commonly seen in patients with COVID-19. A previous case series from Wuhan, China reported 99 confirmed cases of COVID-19, of which 22% had abnormal aPTT, 35% had abnormal prothrombin time (PT), and 36% had increased D-dimer levels [3]. In a recently published brief report from Wuhan, China, the incidence of lower extremity DVT in 81 critically ill COVID-19 patients was 25% [5]. In the case presented, our patient was symptomatic in bilateral upper and left lower extremities, but was coincidentally found to have thromboembolism in all four extremities. Further imaging to rule out a pulmonary embolism was not pursued for the episodic chest pain and tachycardia as she was already on therapeutic doses of enoxaparin. This highlights the importance of having a low threshold to investigate for thromboembolism in hospitalized COVID-19 patients with rising D-dimer levels.

Hypercoagulability workup was pursued in this patient due to the extent of thrombosis and the underlying risk factor of obesity. Obesity has been linked to thrombosis, and a recent study showed that obesity is a risk factor for worse outcomes among patients hospitalized for COVID-19 [6,7]. The coagulopathy workup in our patient revealed equivocal results for lupus anticoagulant antibody testing. It was positive by dRVVT, but negative by PNP. Anticardiolipin IgG and IgM antibodies were positive. In a recent report, three critically ill patients with COVID-19 were found to have multiple cerebral infarctions, and APLA were identified on further investigation [8]. A transient elevation in APLA has also been reported during other viral illnesses from human immunodeficiency virus (HIV) and hepatitis C virus (HCV) [9]. Moreover, severe acute respiratory syndrome (SARS) and Middle East respiratory syndrome (MERS) have also been associated with hypercoagulable states [10]. It is hypothesized that viral syndromes may lead to endothelial dysfunction and trigger an inflammatory response that may result in excessive activation of the coagulation cascade, thereby increasing the risk of thrombus formation [11].

The utility of anticoagulation in COVID-19 patients is not well established. In a recent retrospective analysis of 2,773 hospitalized COVID-19 patients in New York City, a longer duration of systemic anticoagulation was associated with a reduced risk of mortality (adjusted hazard ratio of 0.86 per day, p<0.001). Bleeding events were more common among patients who received systemic anticoagulation (3%) as compared to those who did not (1.9%), but the difference was not statistically significant (p=0.2) [12]. Another retrospective analysis from China looked at the association between 28-day mortality and prophylactic heparin in 449 patients with COVID-19. In the study, 99 patients received low molecular weight heparin for at least seven days; no statistically significant mortality difference was observed between the heparin and non-heparin group. However, in a subset of patients with a sepsis-induced coagulopathy score ≥4 (SIC: consists of platelet count, PT, and Sequential Organ Failure Assessment [SOFA] score) or D-dimer >3.0 mcg/mL, lower 28-day-mortality rates were observed in those who received low molecular weight heparin [13]. Our patient had a SIC score of 3 with significantly elevated D-dimer (17.46 mcg/mL) and improved after being started on anticoagulation and hydroxychloroquine. It is unclear how much hydroxychloroquine contributed to her recovery, as the drug is still undergoing clinical trials and investigation. Further research is needed to investigate the prevalence of thromboembolism and the utility of anticoagulation in COVID-19 patients.

## Conclusions

The present case emphasizes the consideration of DVT and hypercoagulable workup in hospitalized COVID-19 patients with increasing D-dimer levels. As described in our report, the occurrence of thromboembolism may not necessarily be restricted to critically ill patients with sepsis-associated coagulopathic states, such as disseminated intravascular coagulation (DIC) or microvascular thrombosis, and can be attributable to a transient rise in APLA related to a viral syndrome. There should be a low threshold to consider thromboembolism and anticoagulation in hospitalized COVID-19 patients with elevated D-dimer levels.
